# Supplementary material for: The landscape of transcription factor promoter activity during vegetative development in Marchantia
Source: Plant Cell. 2024 Feb 23;36(6):2140–59. doi: 10.1093/plcell/koae053 (PMC11132968; doi:10.1093/plcell/koae053)

Supplemental Figure S1. Variability of the Marchantia gemma dimensions. (Supports Figure 1) (a) Schematic drawing of Marchantia gemma dimensions. (b) Distribution of total length and distance between notches. (c) Distribution of length between notches and gemma border. (d) Correlation between total length and distance from notch to border.

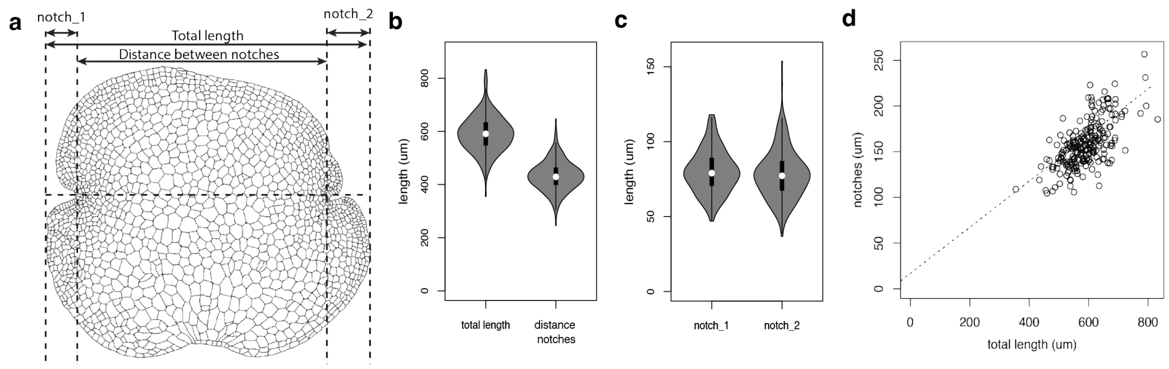

Supplemental Figure S2. (Supports Figure 6) Dynamic activity of additional TF promoters and reporters specific to the SCZ ($_{pro}$Mp*C2H2-26,* $_{pro}$Mp*CDC5,* $_{pro}$Mp*BZIP7*) driving expression of *mVenus-N7 nuclear-localised fluorescent protein*). Cell types of the SCZ are shown on the left. Confocal images of the gene of interest (yellow) and a constitutive plasma membrane marker (magenta, $_{pro}$Mp*UBE2:mScarlet-Lti6b*). Asterisks mark the apical notch. Scale bars = 100 µm. Gene IDs: Mp*C2H2-26* = *Mp8g14220,* Mp*CDC5* = *Mp1g10310,* Mp*BZIP7* = *Mp3g04360.*

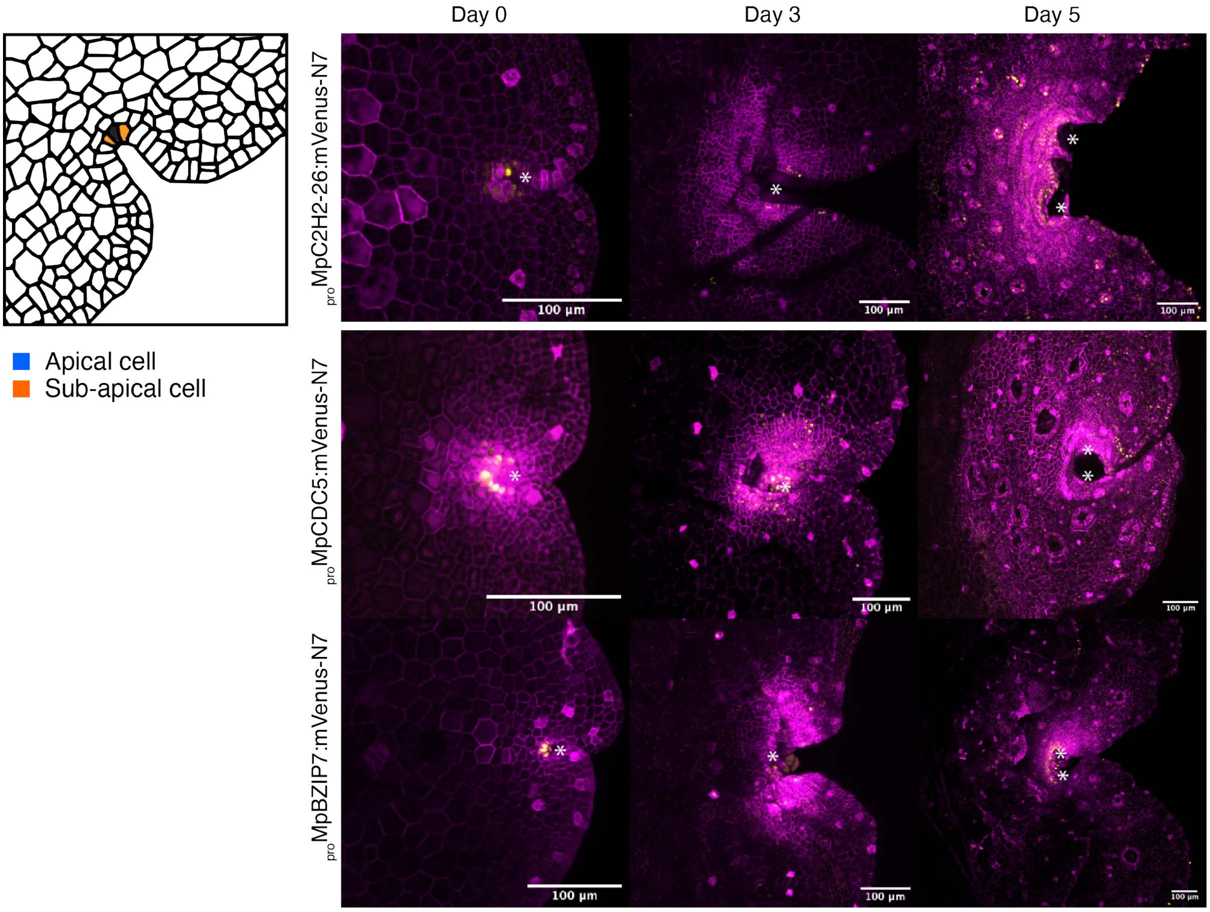

Supplemental Figure S3. (Supports Figure 6) *In situ* localization of Mp*ERF20/LAXR* mRNA in 0-day-old and 3-day-old gemmalings. Signal is specifically localised in cells around the SCZ. Scale bar = 50 μm.

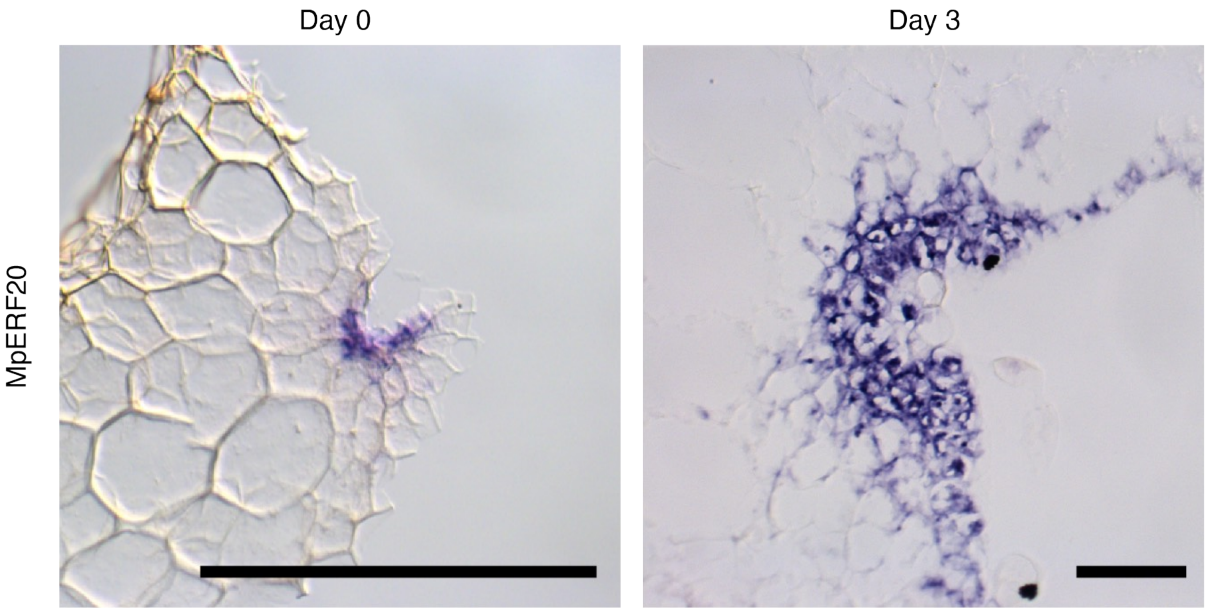

Supplemental Figure S4. (Supports Figure 7) (a) Time-course images of gemmaling development in lines with expression of fluorescent reporters specific to the border (*pro*Mp*ARF2* *pro*Mp*NAC2)*. (b) Time-course images of gemmaling development in lines with expression of fluorescent reporters specific to the attachment (*pro*Mp*ASLBD17*). The attachment and slime papillae are highlighted. Confocal images of the gene of interest (yellow) and a constitutive plasma membrane marker (magenta, *pro*Mp*UBE2:mScarlet-Lti6b*). Scale bar 100 μm. Mp*ARF2 = Mp4g11820,* Mp*NAC2 = Mp6g02590,* Mp*ASLBD17 = Mp8g09250.*

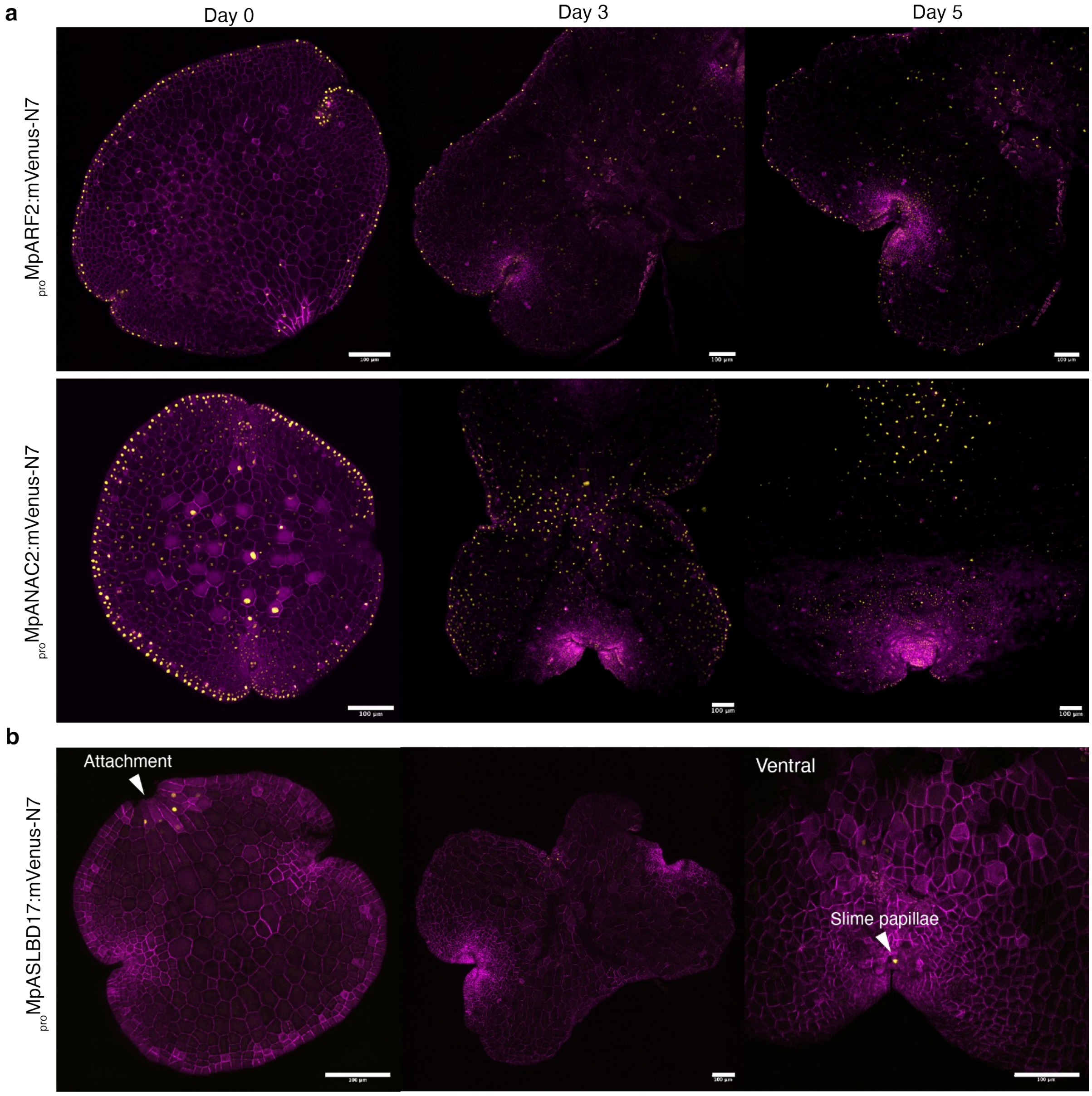

Supplement: koae053_Supplementary_Data [file koae053_supplementary_data.zip › tpc.23.00631Supplemental Figures.pdf]
