# Supplementary material for: The landscape of transcription factor promoter activity during vegetative development in Marchantia
Source: Plant Cell. 2024 Feb 23;36(6):2140–59. doi: 10.1093/plcell/koae053 (PMC11132968; doi:10.1093/plcell/koae053)
Supplement: koae053_Supplementary_Data [file koae053_supplementary_data.zip › TPC2023LSB00631R1_Supplemental_MovieLegend.pdf]

Supplemental Movie S1. **Additional dynamic expression of reporters in the SCZ.** Time-lapse of $_{pro}$Mp*ERF20/LAXR* (yellow) expression after laser ablation of the notches and until re-establishment of the new SCZ. Constitutive plasma membrane marker ($_{pro}$Mp*UBE2:mScarlet-Lti6b*) is shown in magenta.
