# Supplementary material for: The landscape of transcription factor promoter activity during vegetative development in Marchantia
Source: Plant Cell. 2024 Feb 23;36(6):2140–59. doi: 10.1093/plcell/koae053 (PMC11132968; doi:10.1093/plcell/koae053)
Supplement: koae053_Supplementary_Data [file tpc.00631.2023-s07.pdf › tpc.00631.2023-s07.pdf]

THE PLANT CELL

Mapping the landscape of transcription factor promoter activity during vegetative development in Marchantia

Facundo Romani, Susanna Sauret-Güeto, Marius Rebmann, Davide Annese, Ignacy Bonter, Marta Tomaselli, Tom Dierschke, Mihails Delmans, Eftychios Frangedakis, Linda Silvestri, Jenna Rever, John Bowman, Ignacio Romani, and Jim Haseloff

Review Timeline:

Submission Date:	13-Aug-2023
Editorial Decision:	06-Sep-2023
Revision Received:	08-Dec-2023
Accepted:	22-Dec-2023

Prof. Jim Haseloff
University of Cambridge
Cambridge, Cambridgeshire CB2 3EA
United Kingdom

Dear Dr. Haseloff:

We have received reviews of your manuscript entitled "Mapping the landscape of transcription factor promoter activity during vegetative development in Marchantia." Thank you for submitting your best work to The Plant Cell. The editorial board agrees that the work you describe is substantive, falls within the scope of the journal, and may become acceptable for publication, pending revision and potential re-review.

We ask you to pay attention to the following points in preparing your revision:

The editor and reviewers agree that your manuscript is better categorized as a "Large Scale Biology" manuscript since it primarily describes a resource for the Marchantia community and beyond. We have now recategorized your manuscript (noted as LSB in the article designation). In line with this particular category, both reviewers are excited about the work in your manuscript, but we ask you to consider these following:
1. Respond line by line to both referees' concerns
2. To provide experimental data regarding reviewer #1's comment to demonstrate the predictive value of the resource. For instance, this could comprise loss-of-function/misexpression lines for candidate genes to show their function.

Please contact us if there are ambiguous comments or if you wish to discuss the revision.

Given the nature of the comments, we are offering you 60 days from when we have issued this decision to complete the revision. If a revision is not returned within this time frame, and if you have not been granted an extension, we will withdraw the manuscript, which will leave you free to submit the work elsewhere. If you need an extension, we encourage you to contact us at any point before the 60 days have passed.

When you are ready to submit the revised version, please upload a highlighted copy that indicates all changes made in response to the editor and reviewer recommendations. Include an itemized list of all changes made in response to each of the reviewer's suggestions in the "Response to Reviewers" section; please note that reviewers do not have access to your cover letter, nor was this decision letter shared with them.

Thank you for the privilege of reviewing your work. We look forward to receiving your revised manuscript.

Sincerely,

The Plant Cell Board of Editors

Please note the following:

-The Plant Cell now requires authors to complete and submit an author revisions checklist upon submission of a revised manuscript. The aim of the checklist is to aid authors in preparing a high-quality manuscript, facilitate the review and assessment of revised manuscripts, and help to ensure that journal standards are maintained across the board. If your manuscript is accepted, the completed checklist will be published as supplemental material attached to the article online. Please download a copy of the checklist (pdf fillable form) at this link, for submission with your revised manuscript: https://tpc.msubmit.net/html/Author_Revisions_Checklist.pdf.

-Supplemental materials should be restricted to large datasets and tables, presentation of replicates, and validation of reagents, methods, or genotypes. Any data that are used to support the major claims must be in the main manuscript. Supplemental figure legends must indicate what figure in the main manuscript is supported by the supplemental data presented. Please justify how each of the supplemental figures meet the criteria.

-Sampling methods and nature of "biological replicates" should be described precisely (i.e. different plants, parts of plants, pooled tissue, independent pools of tissue, sampled at different times, etc), along with a clear description of and rationale for any statistical analyses conducted. The reader should know exactly what was sampled; what forms the basis of the calculation of any means and statistical parameters reported. This is also necessary to ensure that

proper statistical analysis was conducted.

-Want to add this revision deadline to your calendar? Click below!

--- Reviewer comments:

Reviewer #1 (Comments for the Author):

Romani et al. report a magnificent resource for the Marchantia community, and a rich dataset of expression patterns that helps cement models of development based on classical histology. It also allows to develop new hypotheses regarding tissue ontogeny and cell fate - and of course on gene function. This is a laudable effort that seems open science by design. The patterns and collection of reporters is fully open and available, and I consider this an important, essential resource. At the same time, there are some technical/design aspects that need attention, and the study (for e regular research paper - not a resource) feels a bit "thin" without at least cursory experiments that demonstrate the use of the resource. Specific comments are below:

1. The authors do not explain their rationale for choosing the length of promoters, nor is there any proof of concept that demonstrates the impact that these choices make on the patterns observed. It would be helpful to include (multiple?) alternative promoter lengths for a small set of reporters.

2. The analysis in Figure 5d (clustering analysis of expression domains and dynamics) needs more explanation. What is done here and what does it tell about developmental progression?

3. I was surprised not to see a cluster of cell cycle-regulated patterns. Would some of the non-descript patterns fall into this category? This would deserve exploration.

4. One technical concern is the potential influence of mVenus-N7 protein stability on the patterns observed. Since expression patterns and developmental domains are defined based on reporter fluorescence, it is important to know how (differential?) reporter protein stability contributes to the inference of domains. It would be helpful if - for a small set of reporters - a labile FP were used (e.g. D-box fused to the same protein).

5. While the data presented offers a tremendous resource, it is unclear from the manuscript what its power is in predicting biological function. The data identifies quite a few potential regulators of cell fate or differentiation, and either generating knockouts or misepxression lines for some of these could close the cycle of exploration. I would encourage the authors to include such an analysis, as it would clearly demonstrate that the resource is not only descriptive but also predictive.

Minor:

- Line 227 Figure 4b should refer to Figure 5b
- There seems to be a stub in lines 319 and 320.

Reviewer #2 (Comments for the Author):

The manuscript entitled "Mapping the landscape of transcription factor promoter activity during vegetative development in Marchantia" by Romani et al. demonstrated the comprehensive spatiotemporal patterns of promoter activities of putative transcription factors (PTFs) in Marchantia polymorpha, especially focusing on gemma and gemmalings, which show highly reproducible expression patterns. Developmental patterns (e.g. TZ) and boundaries (e.g. DDCZ/TZ, TZ/PZ) presented here are novel and interesting. These patterns might be regulated by diffusible signals, cell autonomous program, and/or developmental clock. This manuscript is clear and of high quality, providing a new genetic tool.

1. Because fluorescent proteins are generally stable in vivo, they do not reflect transient expression pattern essential for development and patterning. I recommend to acknowledge the limitation of the reporter used here (mVenus-N7).

2. Several (or most of) genes analyzed here are "putative" transcription factors (PTFs), annotated based on the sequence homology, and not studied in the context of transcriptional regulation. Some of them would not be the bona fide transcription factors (for example: C3H family, Mp8g04010). The members of C3H zinc finger protein family do not always function as transcriptional regulators.

3. I recommend to show the reason why about 15% PTF genes exhibited inconsistent expression pattern with RNA-seq (page 6, line 166-167). Are there any features of gene structures (5'/3'-UTR, intron structure, intron position: nonsense mediated decay target) or some favors of gene families?

Responses to Reviewer #1:

Romani et al. report a magnificent resource for the Marchantia community, and a rich dataset of expression patterns that helps cement models of development based on classical histology. It also allows to develop new hypotheses regarding tissue ontogeny and cell fate - and of course on gene function. This is a laudable effort that seems open science by design. The patterns and collection of reporters is fully open and available, and I consider this an important, essential resource. At the same time, there are some technical/design aspects that need attention, and the study (for e regular research paper - not a resource) feels a bit "thin" without at least cursory experiments that demonstrate the use of the resource. Specific comments are below:

1. The authors do not explain their rationale for choosing the length of promoters, nor is there any proof of concept that demonstrates the impact that these choices make on the patterns observed. It would be helpful to include (multiple?) alternative promoter lengths for a small set of reporters.

We thanks the reviewer for raising this point. We added a detailed description of the rationale behind the promoter lengths in the first par of the revised version of the results and in Supplementary Figure S1. This includes references to compare with previous research in other species with Marchantia genomic features. In support, the presence of ATAC-seq peaks can also be used as a reference for the presence of regulatory regions. Despite there is not a formula for defining an ideal promoter length and there are other practical limitations, the compromise that we arrived is 1.8 kb before the TSS and the entire 5' UTR. Based on genomic observations, it is reasonable to think that longer promoters are not likely to contribute major differences in expression patterns.

Empirically, application of this rule of thumb has resulted in a rich collection of active and useful promoter elements. The intention of this work was to create a freely accessible genetic toolkit that would provide cell-specific triggers for transcription. We believe it would be naïve to expect a truncated promoter element to fully recapitulate the dynamics of a source gene's expression in the absence of native distal, 3' proximal, intron, post-transcriptional and post-translational control elements. However, these promoter elements are competent to respond precisely to endogenous transcriptional regulators. They contain native recognition sequences in a compact, standardised format for rapid assembly and reuse, both to allow the facile construction of new genetic circuits, and to provide intrinsic markers for cell types during development. These are tested, and we believe that they are necessary tools for linking the activity of underlying gene regulatory circuits with cellular anatomy and dynamics.

Most of the observed promoter expression patterns are consistent with the evidence of whole tissue RNA-seq, scRNA-seq data for the source genes (see Figure 2). We provide specific examples where the corresponding promoters for some of our synthetic candidates have been described before in the literature (e.g. proMpC1HDZ, proMpERF13, proMpARF1, proMpARF2, and proMpERF20). In these cases, the expression pattern is consistent with previous versions of the promoters despite the fact that our version is shorter.

2. The analysis in Figure 5d (clustering analysis of expression domains and dynamics) needs more explanation. What is done here and what does it tell about developmental progression?

We have revised the text and figure caption to better explain this. The discussion associated

with this aspect is mentioned at line 457-459. Now, in the original version, the figure number was wrong (now corrected).

3. I was surprised not to see a cluster of cell cycle-regulated patterns. Would some of the non-descript patterns fall into this category? This would deserve exploration.

We agree this is a relevant aspects. We carefully checked all expression patterns that we explored in the gemma and other developmental stages, including the ones not specifically described, and non of them could be unequivocally associated with the cell-cycle.

We discuss the correlation between cell division and TF promoter activity patterns in the paragraph between lines 359-384. The DDCZ domain is highly correlated with cells undergoing cell division after germination, but most promoters show a stable expression pattern and do not allow us to distinguish between dividing and non-dividing cells. At the developmental stage when most images were obtained (Day 0), few cells would be dividing. In addition, in Wang et al. (2023) single-cell analysis (that includes other developmental stages) authors were also unable to distinguish between the "meristematic cells surrounding the notch" from actively dividing cells.

Cell cycle dynamics are heavily regulated by targeted protein modification and degradation and this will not be reflected in the observed promoter activity and transcription. As reviewers noted below, the perdurance of the mVenus-N7 marker makes it difficult to track transient expression patterns, if present. We have clarified this point in the text. The promoter collection is useful for visualising transcription dynamics. We agree that further studies of regulation based on protein turnover (such as cell cycle or hormone responses) will require different protein markers, such as the suggested use of D-box reporters, but that is beyond the scope of this study. We mention this issue among the limitations in the Discussion.

4. One technical concern is the potential influence of mVenus-N7 protein stability on the patterns observed. Since expression patterns and developmental domains are defined based on reporter fluorescence, it is important to know how (differential?) reporter protein stability contributes to the inference of domains. It would be helpful if - for a small set of reporters - a labile FP were used (e.g. D-box fused to the same protein).

The use of a stable reporter such as mVenus-N7 presents some constraints. We have modified the text to highlight the potential effect of protein stability on interpretation of observed transcription patterns. The reason for using such a marker is to identify stable cellular states. Use of a stable fluorescent protein reporter is a standard approach for developmental biology in plants. Most other toolkits for Arabidopsis promoters and fluorescent proteins have been used without degradation tags (Schurholz et al., 2018 Plant Journal). The use of a destabilised gene product would potentially allow better accounting for transient changes in transcription rates, but at the expense of sensitivity, and with the introduction of additional potential sources of biological variation. There is the additional complication of choosing which destabilising principle to use in Marchantia, in the absence of a widely accepted standard.

As it stands, the mapping of expression domains is based on comparative analysis of hundreds of promoters with different strengths, which also account for biological variation in the delimitation of the domains. In future work, the use of labile FP variants may be useful for tracking short-lived cell states such as during cell division or environmental responses. We suggest that a functional cellular map such as the one presented in the manuscript is a necessary prerequisite for finer scale studies that would account for additional layers of post-

transcriptional/post-translational regulation in the future. Further, the associated promoter elements will provide the starting materials for these future studies.

5. While the data presented offers a tremendous resource, it is unclear from the manuscript what its power is in predicting biological function. The data identifies quite a few potential regulators of cell fate or differentiation, and either generating knockouts or misepxression lines for some of these could close the cycle of exploration. I would encourage the authors to include such an analysis, as it would clearly demonstrate that the resource is not only descriptive but also predictive.

The main intention of this work has been to construct a comprehensive library of promoter elements that are (i) show a wide range of cell specificities, (ii) are compact and easy to reuse in new DNA circuits, (iii) and allow us to better map cellular microarchitecture with underlying patterns of gene regulation. We have successfully used the proximal domains of TF genes as a rich pool of synthetic promoter activities. The properties of the promoters were tested directing by fusion to a reporter gene and screening in vivo, and have proven robust in independent experiments.

Primarily, a researcher interested in mapping or reprogramming gene expression has a new, comprehensive set of tools to work with. As we agree with the recategorisation of the manuscript as Large Scale-Biology manuscript, the resource aspect is the main strength of the paper.

Secondarily, expression patterns can also provide important clues to the biological roles of transcription factors. This fact does not require a demonstration, since it is widely a concept widely accepted in developmental biology. So the toolset can provide the geneticist with many interesting candidates to explore. During the text, we do not draw conclusions about biological function of particular TFs. Yet, the predictive potential of the resource can be assessed by comparing our observed expression patterns with existing functional studies.

During the screening we noted cases where the expression pattern of a promoter correlated with known source gene function. Clear cases were found for oil bodies, where MpC1HDZ (Romani et al., 2020 Curr Biol), MpERF13 (Kanazawa et al., 2020 Nat Comms) and MpMYB2 (Wang et al., 2023 Dev Cell) are specifically expressed in oil body cells and mutant plants showed developmental defects in the same cell type. The functional characterisation of MpMYB02 in oil body development was published practically at the same time that we released this manuscript. In our hands, the Mpmyb02 loss-of-function also showed the same phenotype.

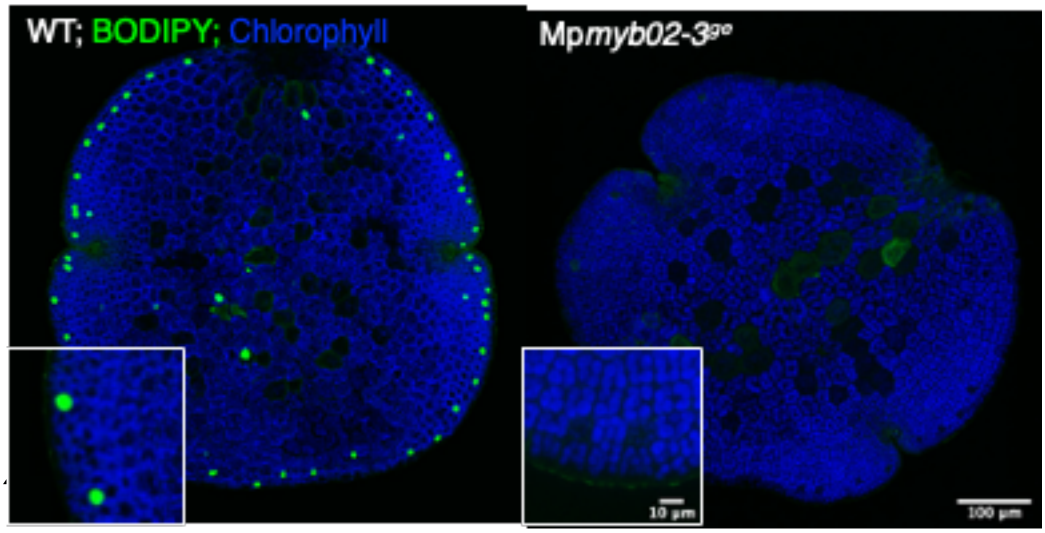

Since this has been published in Want et al. (2023, Dev Cell), adding such results will not contribute any novel insight. Thus, during promoter screening, we 'rediscovered' these genes that were identified due to their distinct patterns of expression in oil cells.

Further, we showed that the promoter derived from MpERF20 drives expression in the apical cell. Functional studies of MpERF20 have demonstrated that the TF activity is both required and sufficient to generate new meristems during regeneration (Ishida et al., 2022, PCP). We have now provided a new marker, which we have used for time-lapse and in-situ hybridisation to confirm the association with meristem stem cells and regeneration. We have cloned a longer version of the proMpERF20 reporter and confirmed that the promoter expression patterns are consistent. We have better highlighted these examples in the text.

A further clear example of the utility of the screen is from Frangedakis et al. (bioRxiv, 2023), where the promoter of MpRR-MYB5 described in the current work was used to functionally complement mutant lines. In addition, we have successfully functionally complemented MpGLK and MpBHLH12 mutants (unpublished data).

There are many other candidate promoters in this study that may provide strong indications of the role of the cognate TF. Unlike the promoter properties themselves, the expected TF phenotypes are not easy to predict and require analysis that are unfeasible for the time given for this revision. We believe that this resource for functional studies is a major strength of the work and have emphasised this in the text.

Minor:
- Line 227 Figure 4b should refer to Figure 5b
- There seems to be a stub in lines 319 and 320.
These have been corrected.

Responses to Reviewer #2:

The manuscript entitled "Mapping the landscape of transcription factor promoter activity during vegetative development in Marchantia" by Romani et al. demonstrated the comprehensive spatiotemporal patterns of promoter activities of putative transcription factors (PTFs) in Marchantia polymorpha, especially focusing on gemma and gemmalings, which show highly reproducible expression patterns. Developmental patterns (e.g. TZ) and boundaries (e.g. DDCZ/TZ, TZ/PZ) presented here are novel and interesting. These patterns might be regulated by diffusible signals, cell autonomous program, and/or developmental clock. This manuscript is clear and of high quality, providing a new genetic tool.

1. Because fluorescent proteins are generally stable *in vivo*, they do not reflect transient expression pattern essential for development and patterning. I recommend to acknowledge the limitation of the reporter used here (mVenus-N7).

Reviewer #1 also raised this point. We have addressed this limitation more clearly in the Discussion section of the manuscript as detailed above.

2. Several (or most of) genes analyzed here are "putative" transcription factors (PTFs), annotated based on the sequence homology, and not studied in the context of transcriptional regulation. Some of them would not be the bona fide transcription factors (for example: C3H family, Mp8g04010). The members of C3H zinc finger protein family do not always function as transcriptional regulators.

We have modified the text following the reviewer's suggestion.

3. I recommend to show the reason why about 15% PTF genes exhibited inconsistent expression pattern with RNA-seq (page 6, line 166-167). Are there any features of gene structures (5'/3'-UTR, intron structure, intron position: nonsense mediated decay target) or some favors of gene families?

Our view is simply that the reporters capture will capture features of the transcriptional regulation of their cognate putative TF genes. The most likely explanation for any inconsistency between promoter activity and native expression (e.g. reflected in RNA-seq data) is potential absence of some regulatory elements upstream (or downstream) of the gene as discussed on lines 411-420. For example, a version of proMpSHI, showed expression when using a longer promoter with a GUS reporter (Flores-Sandoval et al., 2015 PLOS Genetics). In another example, MpKNOX1, we observed promoter activity but the RNA-seq showed the opposite, likely due an upstream anti-sense transcript called MpSUK1 (Dierschke et al., 2021 eLife).

We identified a couple of cases where the inconsistency could be explained by the presence of introns and alternate start codons. For example, in the MpBHLH45 gene, there is an intron and two splice variants with different start codons. Since we used the Mp2g19970.1 variant as the reference for the promoter, the first intron was not included in the promoter. That region, part of the 5UTR of Mp2g19970.2, might contain regulatory regions that are missing.

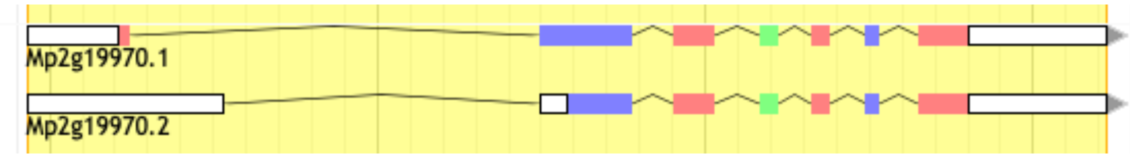

Similar is the case of Mp1g26740 (MpTRIHELIX5),

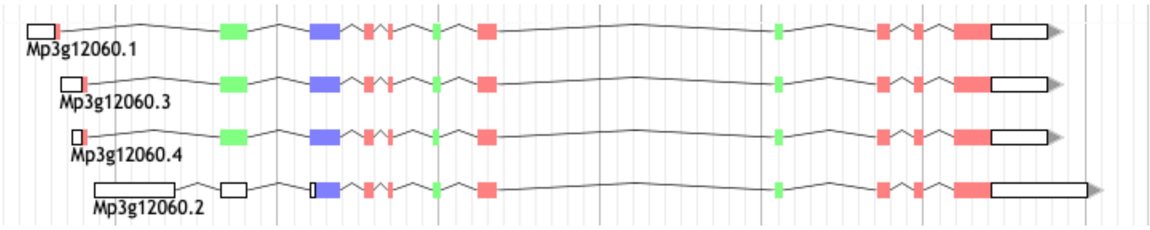

Mp2g26400 (MpBHLH49).

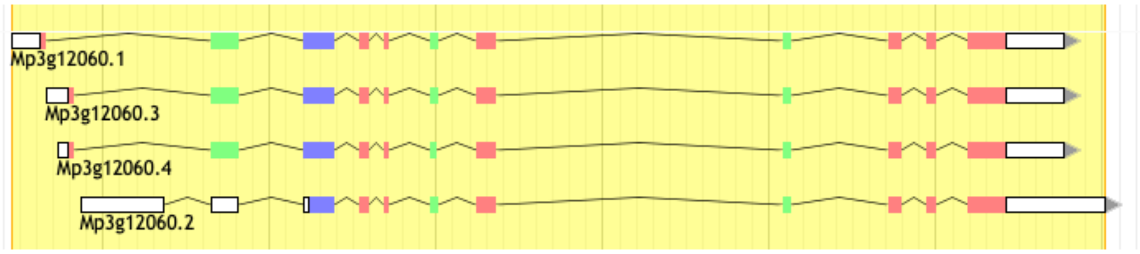

We have modified the text to raise these potential issues and awareness of the effects of potential annotation artifacts in line 420-421.

Dear Dr. Jim Haseloff:

We are pleased to inform you that your paper entitled "Mapping the landscape of transcription factor promoter activity during vegetative development in Marchantia" has been accepted for publication in The Plant Cell, pending a final editorial review by a science editor. At this stage, your manuscript will be evaluated by a science editor with respect to its presentation of scientific content, compliance with journal policies, and presentation for a broad readership. The Plant Cell has contracted with Plant Editors (planteditors.com) to provide this service to our authors, and you will soon receive additional information on this process.

Please note that each author needs to link their ORCID identifier to their account in the system before your manuscript can be published. If any authors do not have an ORCID linked to their account, they will receive a message with a link to complete this task. Please ensure that ALL of your coauthors have completed this task as soon as possible.

ASPB offers an OPEN option that allows authors to have their online articles available for free to all users immediately upon publication. For more information about the ASPB OPEN option, refer to the Final Submission Checklist Form.

The Plant Cell and The Arabidopsis Information Resource (TAIR) are collaborating to collect functional annotation data about Arabidopsis genes from authors. This includes information about the gene's molecular function (e.g., kinase activity, ATP synthetase activity), the biological process/es it is involved in (e.g., endosperm development, threonine biosynthesis), its subcellular location (e.g., nucleus, ER), anatomical or developmental expression pattern (e.g., leaf, ovule, flower stage 10, seedling stage), or its partner in a protein-protein interaction (e.g., AT1G01010 interacts with AT1G01020).

If your paper contains results falling into one or more of these categories for Arabidopsis genes, we request that you now submit these data for inclusion in TAIR by filling in the form provided at the following URL: https://www.arabidopsis.org/doc/submit/functional_annotation/123. If you need further clarification on what types of data can be submitted please contact curator@arabidopsis.org.

Finally, we encourage your submission of artwork for the journal cover. Monthly issues will have an online cover image and selected covers will be used for posters, other promotional items, and "wallpaper" for mobile devices. For more information, refer to Cover Submission in the Instructions for Authors https://tpc.msubmit.net/cgi-bin/main.plex?form_type=display_auth_instructions.

We look forward to seeing your paper published.

Sincerely,

The Plant Cell Board of Editors

=====
IMPORTANT REMINDER: PEER REVIEW REPORTS
=====

If you opted to publish a peer review report along with your article during the original submission process, it will be prepared by the editorial staff and publicly posted with your manuscript, inside the zip file that contains any other supplemental material. As a reminder, the peer review report is a public record of all comments from editors and reviewers, as well as your prior responses, as you received them in the decision letters for each draft of your manuscript. If you agreed to publish this report and have changed your mind, or are not sure if you selected this option, please contact the editorial office as soon as possible before signing the license agreement from our publisher.

=====
REVIEWER COMMENTS
=====

Reviewer #1 (Comments for the Author):

The authors have made textual changes to the manuscript to deal with some of the comments I raised. While I can agree with the content of their responses, I am not overwhelmed. It would have been realistic and not unreasonable to:

1. Use different promoter fragments for one or a few genes to test the impact of promoter length on the pattern(s)
2. Compare unstable reporter with the stable one used for one or a few promoters to determine the impact of FP stability
3. Report on phenotypes of one or a few mutants for newly discovered TF's with interesting patterns.

Only the latter is indirectly done through citing papers that have recently appeared.

I remain of the opinion that this is a potentially very useful, but essentially non-validated resource. Even for an LSB paper, I would expect some degree of validation or exploration of limitations. Without this, it is close to impossible to define utility of the resource.

Reviewer #2 (Comments for the Author):

The authors have well addressed comments and questions.
I have no objection and no hesitation in recommending publication in this journal.

----FOR ASPB OFFICE USE ONLY (DO NOT EDIT)----
MSID: 35374
Corresponding Author: Prof. Haseloff
